# How an urban Aboriginal and Torres Strait Islander primary health care service improved access to mental health care

**DOI:** 10.1186/s12939-015-0183-x

**Published:** 2015-06-06

**Authors:** Julie Hepworth, Deborah Askew, Wendy Foley, Deb Duthie, Patricia Shuter, Michelle Combo, Lesley-Ann Clements

**Affiliations:** School of Public Health and Social Work, Queensland University of Technology, Brisbane, Australia; Southern Queensland Centre of Excellence in Aboriginal and Torres Strait Islander Primary Health Care, Brisbane, Australia; Oodgeroo Indigenous Support Unit, Queensland University of Technology, Brisbane, Australia

**Keywords:** Access, Cultural safety, Indigenous, Aboriginal and Torres Strait Islander, Mental health services, Primary health care, Integrated care, Qualitative research, Psychology, Social work

## Abstract

**Introduction:**

Aboriginal and Torres Strait Islander people experience higher levels of psychological distress and mental ill health than their non-Indigenous counterparts, but underuse mental health services. Interventions are required to address the structural and functional access barriers that cause this underuse. In 2012, the Southern Queensland Centre of Excellence in Aboriginal and Torres Strait Islander Primary Health Care employed a psychologist and a social worker to integrate mental health care into its primary health care services. This research study examines the impact of this innovation.

**Methods:**

A mixed-method research design was used whereby a series of qualitative open-ended interviews were conducted with 7 psychology clients, 5 social work clients, the practice dietician, and the social worker and psychologist. General practitioners, practice nurses, Aboriginal Health Workers and receptionists participated in 4 focus groups. Key themes were identified, discussed, refined and agreed upon by the research team. Occasions of service by the psychologist and social worker were reviewed and quantitative data presented.

**Results:**

Clients and staff were overwhelmingly positive about the inclusion of a psychologist and a social worker as core members of a primary health care team. In one-year, the psychologist and social worker recorded 537 and 447 occasions of service respectively, and referrals to a psychologist, psychiatrist, mental health worker or counsellor increased from 17 % of mental health clients in 2010 to 51 % in 2012. Increased access by Aboriginal and Torres Strait Islander people to mental health care was related to three main themes: (1) *Responsiveness to community needs*; (2) *Trusted relationships*; and (3) *Shared cultural background and understanding*. The holistic nature and cultural safety of the primary health care service, its close proximity to where most people lived and the existing trusted relationships were identified as key factors in decreasing barriers to access.

**Conclusions:**

Improving social and emotional well-being is critical to addressing the health inequalities experienced by Aboriginal and Torres Strait Islander peoples. This study demonstrates the benefits for clients and health professionals of integrating culturally safe mental health services into primary health care.

## Introduction

The continuing effects of colonisation, intergenerational trauma, and widespread social and economic disadvantage have contributed to the poorer health status of Aboriginal and Torres Strait Islander peoples compared to their non-Indigenous counterparts. Additionally, experiences of institutional racism have presented, and continue to present, a challenge in terms of engaging with health services, particularly mental health services [1, 2]. Ironically, these very issues also render Aboriginal and Torres Strait Islander peoples vulnerable to mental ill health [3]. Mental ill health has been estimated to make the second highest contribution (15 %) to disease burden in the Indigenous population accompanied by a disproportionally high level of unmet needs [2, 4] and further exacerbated by disproportionally low access to mental health services [5, 6]. Highlighting this gap, Fielke [7] observed; “*Indigenous people do not enter mainstream care expecting to be treated well*” (p.S75).

As a result of reluctance to use mainstream mental health services, many Indigenous people do not receive early intervention and/or preventative care. Consequently, responses to Indigenous mental ill-health are frequently reactive and in response to crises, and at times may also involve the police [4, 8]. This situation results in a cascade of negative effects precipitated by misunderstanding and miscommunication – a negative experience for both staff and patients [4]. Such past failures, experiences of stigma and racism and a fear of being incarcerated perpetuate reluctance to access mainstream mental health care services within the community [4, 9].

The process of integrating mental health workers into Indigenous primary health care teams has been the subject of consideration by researchers and policy makers [7, 10–12]. Indigenous people have been shown to be more likely to visit an Indigenous mental health worker, and especially one who is highly visible in the community [7, 10]. Nagel [2] emphasises the imperative for mental health workers to focus on strengths based interventions and protective factors, with the objective of recovery for Indigenous clients. The National Aboriginal and Torres Strait Islander Health Plan: Key strategies for mental health and social and emotional well-being [13] acknowledges the holistic nature of social and emotional well-being (SEWB) and the need to support strategies that promote culturally safe services that integrate SEWB in all aspects of health care delivery for Indigenous communities. This research study examined the impact of integrating mental health care service delivery into an urban Aboriginal and Torres Strait Islander primary health care service.

## Methods

### Aboriginal and Torres Strait Islander community approval and ethical clearance

This study included a commitment to conducting research within an appropriate ethical framework as recommended by the National Health and Medical Research Council’s Values and Ethics – Guidelines for Ethical Conduct in Aboriginal and Torres Strait Islander Health Research [14]. Ethical research conduct involved two key processes. The Inala Community Jury for Aboriginal and Torres Strait Islander Health Research (a group of local Aboriginal and Torres Strait Islander people who guide all research undertaken by the Southern Queensland Centre of Excellence in Aboriginal and Torres Strait Islander Primary Health Care (Centre of Excellence) provided community support for the project [15]. Ethical clearance was obtained from the Metro South Human Research Ethics Committee. Results were disseminated back to the Inala Aboriginal and Torres Strait Islander community via the Community Jury at completion of the project and to the Centre of Excellence staff at a staff forum.

### Setting

The Centre of Excellence (formerly known as the Inala Indigenous Health Service) is a Queensland Government general practice located in Inala, a South-Western suburb of Brisbane 18 km from the Brisbane central business district. In 2011 Aboriginal and Torres Strait Islander people made up 6.6 % of the population (910/13,796), significantly more than the national proportion of 2.5 % in the same year [16]. The Centre of Excellence has a long history of improving access to care, with an original practice population of 12 Aboriginal and Torres Strait Islander people in 1995 to approximately 10,000 adult patients in 2014, the majority of whom are Aboriginal and/or Torres Strait Islander who reside in Inala or surrounding suburbs [17].

In 2011, the Centre of Excellence received funding from the Queensland Government to initiate a program of activity aiming to improve the prevention, management and treatment of chronic disease. In recognition of the significant impact of chronic disease on mental health and SEWB, and the history of access barriers to culturally appropriate mental health care services, funding was allocated to employ a psychologist and social worker as core members of the primary health care team. In response to community feedback, we specifically targeted Aboriginal or Torres Strait Islander providers to fill each position.

### Study design

The study was based on a mixed-method research design including two components: (1) descriptive statistics on service access, and (2) a qualitative research approach [18]. We used a predominantly qualitative type of mixed-method design, a QUAL-*quan* [19], because of the paucity of existing research about the provision of mental health services for Aboriginal and Torres Strait Islander people in urban primary health care. This research design included the use of interview schedules with broad, open-ended question areas to inform a conversational style of data collection with both service clients and members of the primary health care team. Consequently, the identification of key themes in the interviews that reflected participants’ perspectives was achieved rather than pre-defined categories being imposed by the researchers onto the qualitative data. Given the large amount of qualitative data generated, this paper presents results about a specific area - the impact of the inclusion of a psychologist and social worker in a primary health care service on *access to mental health services* by Aboriginal and Torres Strait Islander people.

### Participants, sampling and recruitment

The study included two groups of participants: (1) clients of the psychologist and/or the social worker, and (2) members of the primary health care team. Clients of the psychologist and social worker were eligible if neither of these two health care providers had provided active care within the 3 months prior to the research commencing and the health care providers considered they were socially and psychologically able to participate in the research. Purposive maximum variation sampling [18] was used to ensure inclusion of a range of clients seen by each health care provider (seven clients of the psychologist, five clients of the social worker, including two clients of both the psychologist and social worker), age (6–67 years, although the parent of the 6 year old was the interviewee), gender (four men, eight women), and referral type, referral condition and the number of appointments (14 appointments (*n* = 1), six appointments (*n* = 2), five appointments (*n* = 1), four appointments (*n* = 1), and three appointments (*n* = 3). To minimise selection bias, lists of potential client participants were prepared by the social worker and the psychologist to cover each parameter, and then one of the non-clinical research team members selected potential participants in each category. Based on a recommendation by the Community Jury, the psychologist and social worker telephoned the selected clients, gauged their well-being, informed them about the research and gained verbal consent for a researcher to contact them about participation in the research. Clients were then contacted by a researcher to further discuss the research, and, if agreeable, to arrange a mutually convenient time for an interview. The primary health care team participants included general practitioners (GPs), practice nurses (PNs), Aboriginal health workers (AHWs), receptionists, the dietician, the psychologist and the social worker. All team members were employees of the Centre of Excellence, eligible to participate, and actual participation was determined by availability at the times when the interviews occurred.

### Data collection and analysis

1. Descriptive statistics on service access

The quantitative data identify the total number of occasions of service of the social worker and the psychologist, the number of referrals to the psychologist being made by the GPs, and the number of general practice mental health care plans commenced. To determine changes in clinical practice, we compared the proportion of mental health clients being referred to a mental health worker, counsellor, psychologist or psychiatrist in the year preceding the employment of the psychologist and the social worker (2010) and in the year after their employment (2012).

(2) Qualitative research

An Aboriginal member of the research team who was not an employee of the Inala Indigenous Health Service conducted the client interviews where possible in order to protect clients’ privacy, and to ensure maintenance of clients’ cultural safety. When this was not possible, interviews were undertaken by a non-clinical member of the research team with experience of conducting qualitative research in this community. Interviews took place in the clients’ homes (*n* = 3), at the health service (*n* = 7) or via the telephone (*n* = 2). Participants were each thanked with a AUD$25 gift card. Each interview aimed to cover similar broad areas including questions about their perceptions of the work of the social worker and/or psychologist, experiences of accessing a social worker and/or psychologist prior to the employment of these health professionals at the Centre of Excellence, expectations and experiences of appointments with the social worker and/or the psychologist at the Centre of Excellence, and suggestions for changes or improvements to these service. Further recruitment of participants was not required after the completion of 12 interviews because data saturation was achieved.

Four focus groups (GPs *n* = 10; PNs *n* = 6; AHWs *n* = 5; receptionists *n* = 2) and one interview with the dietician were held with members of the primary health care team with each interview lasting approximately 35 min. The focus groups and the dietician interview covered expectations of services provided by the social worker and/or psychologist, the impact of having the social worker and the psychologist as core members of the primary health care team, past and current referral processes for mental health care, patient outcomes resulting from the increased access and availability of the social worker or psychologist, and suggestions for improvements. Interviews with the psychologist and social worker covered expectations of their role, referrals, working inter-professionally and with other agencies and suggestions for how the service could be shaped in the future. A non-clinical member of the research team facilitated the focus groups and conducted the interviews in an attempt to minimise observer bias.

The interviews and focus groups were digitally audio-recorded with permission, transcribed by a transcription service, and data were checked and de-identified prior to analysis. The only transcript corrections required pertained to the use of Aboriginal English. Mainstream English speakers often misunderstand this dialect of English, used consistently in the Aboriginal and Torres Strait Islander community, and these misunderstandings were evident in the transcriptions. First, each interview was summarised by two members of the research team allowing for the independent identification of key themes and sub-themes that were subsequently discussed with all members of the research team over a series of four meetings. During these discussions about the analysis, the key themes were compared across all interviews until the major and sub-themes were agreed upon by the research team. An important part of the analysis was the involvement of the social worker and psychologist who provided clarification about their roles and responsibilities and increased the accuracy of reporting the relationship between the themes and service delivery. Illustrative extracts from each of the themes were selected by the research team and are presented in the next section to demonstrate key issues voiced by participants and to ensure a range of perspectives. The results about access are reported in this paper.

## Results and discussion

1. Descriptive statistics on service access

In the first 12 months that the social worker and psychologist were employed by the Centre of Excellence they recorded 447 and 537 occasions of service, respectively. GPs referred 226 clients to the psychologist, and 199 mental health care plans were commenced. Referrals to the social worker were not recorded in the practice management software as she did not have a Medicare provider number at the time of this study and therefore this information was not available for this research. In 2010, 17 % of mental health clients were referred to a psychologist, psychiatrist, mental health worker or counsellor external to the service, increasing to 51 % of mental health clients in 2012, after the psychologist and social worker were employed at the service.

(2) Qualitative research

The qualitative data demonstrates three key themes about Aboriginal and Torres Strait Islander people’s access to, and health service staff experiences of, mental health care services within a primary health care service. The three themes are: (1) *Responsiveness to community needs*; (2) *Trusted relationships*; and (3) *Shared cultural background and understanding*.

### Theme 1. Responsiveness to community needs

A key way in which clients and staff talked about receipt of mental health care at the Centre of Excellence was to contrast the service with mainstream mental health services. Several problems that clients and staff had previously faced are described as well as what had improved and why since the inclusion of the psychologist and social worker within the Centre of Excellence primary health care team. All participants reported improved access and dramatic changes in the nature of mental health care that was now available through the Centre of Excellence.

#### Mental health care at the Centre of Excellence versus mainstream services

Prior to the employment of the psychologist and social worker at the Centre of Excellence, there were several access barriers for service users. In particular, although publicly funded mainstream services were available, clients had to travel approximately 16kms to access them. Both clients and health professionals identified that local services were needed in order to remove the significant logistical and attitudinal obstacle of having to travel away from their secure environment, especially at times of crises.

If I had to travel across town, I probably wouldn’t have done it. That would have just been [*impossible*]– well, at that point in time (Woman, 53 years old).

Also, in using the mainstream system there was no cultural connection and service users shared the view that seeing an Indigenous health care provider was important.

I was seeing other psychologists outside the services and I went through about 10 of them. Didn’t click. So I thought it might have been a cultural difference (Woman, 43 years old).

Similarly, health service staff described mental health services in Queensland as generally being a negative experience for Aboriginal and/or Torres Strait Islander peoples because of the geographic, bureaucratic and cultural barriers to accessing appointments, a lack of understanding about Indigenous culture and SEWB - especially that much of the work involved service users presenting in crises - and the more general limitations of the mainstream mental health system, such as a lack of communication and integration among health care providers.

I think mental health in Queensland is poorly done. Access to services for Aboriginal people is pretty pathetic (GP: Focus group).

There have definitely been lots of individual reports from people about how they might have been referred to psychological services … in the past but never actually went for reasons: comfort talking to another Indigenous person; no local clinic; payment … stigma and misunderstanding (Psychologist: Interview).

#### Holistic, culturally appropriate care

In contrast to mainstream mental health services, staff described inclusion of mental health services within the primary health care team at the Centre of Excellence as enabling the provision of “holistic” and “culturally appropriate” care, increasing accessibility, and having a positive impact on service users.

[*The Centre of Excellence provides*] holistic care. There’s one young fellow came in quite disturbed and then I referred him to the social worker and then just seeing him about a month down the track, smile on his face. And so the system does work and I just was thrilled about holistic care because I’ve worked in a lot of places and all (PN: Focus group).

They do a lot better than – in my opinion, than the mental health mainstream system does because they’re easily accessible and they are culturally appropriate. The turnover is quite good. I think within a week you can see [*someone*] (AHW: Focus group).

There was a different type of atmosphere working with our community, with your own community. Like I know a lot of clients from a family perspective … and still have that professional boundaries (Social Worker: Interview).

An unexpected consequence of the inclusion of the mental health services into the core business of the Centre of Excellence was referrals by other agencies, particularly government agencies, of Aboriginal and Torres Strait Islander people to the psychologist or social worker. However, these referrals tended to occur without communication and a lack of awareness about existing client load or local practice in treatment plan development. Consequently, these agencies have required education about the roles and clinical processes of the social worker and psychologist, and clarification of the differences between their therapeutic roles and the roles of the referring agencies:

I say to [*patients*] ‘[*The agencies*] can refer you to our service and they can refer you to our clinic or my name on that plan but not actually tell me or ask me … but once you come here, how often we meet and what we talk about is up to us’. If [*the agencies*] tell someone they’ve got to come weekly I will decide whether I need to see them weekly or not and I give [the agencies] that feedback and they often accept it (Psychologist: Interview).

The contrast between mainstream (or external) mental health services and the Centre of Excellence is very pronounced whereby the former is reported to have multiple access barriers for Aboriginal and Torres Strait Islander people. This finding is consistent with existing research that has identified a range of challenges for Indigenous peoples accessing mental health services [1, 2], and in turn the lack of appropriate services contributes to worsening mental ill health [3]. The integration of a psychologist and social worker within the Centre of Excellence in ways that were responsive to the needs of Aboriginal and Torres Strait Islander people created mental health services that were delivered differently. This was based in part on the shared understanding that what was required of the service was distinctly different from mainstream services and would remain negotiated and responsive to service users.

### Theme 2. Trusted relationships

Service users described their experience of accessing mental health care services at the Centre of Excellence in terms of the importance of supportive relationships and feeling “comfortable” in the environment.

The whole Inala [*Indigenous*] Health [*Service*], Murri Health, they’re so approachable, and the nurses. Somebody should do something big for them. They’re just beautiful, comfortable, and it’s so caring that it’s not – I can’t think of the word for that, not feel sorry for you (Woman, 43 years old).

Health service staff talked enthusiastically about the strong relationships that underpinned the care provided by the Centre of Excellence, and attributed increased access to mental health services to several relational features. Importantly, patients of the Centre of Excellence were already familiar with the longstanding Aboriginal and Torres Strait Islander primary health care service and they had existing positive and trusting relationships with the health service staff that enabled greater ease of access. The clinic staff regularly referred to these trusting relationships as the following extracts illustrate:

There’d be elements of trust, where they trust the psychologist because they’re under our roof – I think there’d be elements of just the access where they know where it is and they know how to get there and they’re comfortable in that surroundings (GP: Focus group).

It comforts the patient too to know that that’s a one-stop shop and they don’t have to go outside, because so many patients feel comfortable in our service (Dietician: Interview).

The staff explained how the dynamics of the relationships between health service staff and service users was pivotal to ongoing care and these relationships facilitated and promoted the provision of effective health care.

Also, [*the social worker and psychologist are*] quite – they’re full-time so it’s not something [*clients*] have to wait around for either, so it’s – they get in quite quickly and they can build a relationship because they’re there all the time, so they don’t have to go from one to the other and then, I guess, the thing is when people have to travel outside of Inala, the chance of them going isn’t always quite high (AHW: Focus group).

More freely [*refer*] – often there’s lots of issues. People never come in with one – with their sore foot or whatever. They might have the sore foot but there’s all the other stuff that goes on. So you could actually talk about that and say that we have these services but if you’re thinking, trying to refer that externally that just wouldn’t work (PN: Focus group).

While previously referrals had been made to external mental health services these had not worked well, and in many instances clients had simply not attended. The psychologist gave an example of a conversation she had had with a male client who she and the community considered to be an Elder who had told her that he and those he knew would not attend anywhere else. She stated that his message was clear; *“If you don’t continue don’t refer us to anyone because we are not going to go”*. The fact that clients were comfortable using the service was also recognised by the reception staff, and when the staff were asked about potential stigma resulting from seeing a psychologist they did not see clients as being concerned.

I think they appreciate it, being so close and not having to travel … I don’t think – no, I don’t think anyone’s worried about going to see the psychologist (Receptionist: Focus group).

Here, the participants have illustrated the relational dynamics involved in building trust and its relationship with increasing access. As Fielke et al. [7] argue, Indigenous people do not expect to be treated well entering services, which in turn, highlights the ways in which the relational aspects of the mental health care at the Centre of Excellence functioned to enable and maintain access sufficiently for clients to obtain advice and support.

### Theme 3. Shared cultural background and understanding

The significance of shared cultural understanding was important for the clients. As mentioned above, the psychologist and social worker were both Aboriginal, and therefore clients felt a sense of comfort and familiarity that contributed to the development of a therapeutic relationship. This was particularly important given the feelings of shame associated with mental health issues in the community.

#### Shared cultural identity

From the perspectives of service users being able to identify with a health care provider who was Aboriginal and/or Torres Strait Islander had positively contributed to improving service access and its effectiveness. In both extracts below, provided by a woman and a man, it was the experience of the social worker and the psychologist being Aboriginal that had enabled the clients to obtain help because of the shared understanding of the similar issues that they faced.

I think it helped that she [*the psychologist*] was a black woman … young and Aboriginal … because we have this – deal with the same issues and stuff. I don’t know her background and stuff but as black women, we get dealt with the same stuff in everyday society (Woman, 29 years old).

She knew where we were coming from, ‘cause she’s an Aboriginal lady too, like – so she knew half the problems and that and where I was coming from and even with the kids and stuff like that (Man, 49 years old).

The cultural significance of the psychologist and social worker having Aboriginal backgrounds cannot be overestimated. In the extracts directly below, as in other extracts presented in the analysis under related themes, the connectedness of service users and health service providers through their Aboriginality was clearly beneficial to accessing the service and its therapeutic effectiveness.

Having Aboriginal and Torres Strait Islander staff outstandingly builds trust – there is a risk for mainstream services to not really understand the barriers or the difficulties that people face in their life – they think they are going to get judged … and so it has outstandingly overcome those barriers of access (GP: Focus group).

Often they’ll know a relative of the social worker so, straight away, they feel that connection and they’re happy to go – because, definitely having somebody of Aboriginal background in the social worker or psychology role is, I think in the service, really crucial, so that’s often that turning point for people in that decision-making process (Dietician: Interview).

Although, having a shared cultural background was not unproblematic. Other health care staff discussed very different views about a shared cultural background in terms of the fears held by some service users that their mental health issues may become known within their own communities.

I’ve got a girl at the moment who’s got postnatal depression and she said that she didn’t want to know the psychologist. “I don’t want – I’m Murri [an Aboriginal person from Queensland]. I’m Murri”. She didn’t want a Murri, because that just conflicts with her and the family members might find out (Nurse: Focus group).

Here, the concern held by some service users that their privacy might be compromised if they consulted an Aboriginal health service provider is clear. Aboriginal peoples value an extended family system, and family is an integral part of an Aboriginal person’s life. These extensive networks can contribute to concerns about a lack of privacy and sharing of personal information which could mean that family members become aware of mental health problems.

#### Shame and mental health

Health service users also identified a fear that other community members might find out about another family member’s mental health and/or social problems. Even though the decision to physically locate the psychologist and social worker in a different building from the general practice clinic was due to a lack of available space in the clinic and not intended to provide a more private space both service users and staff recognised that its location contributed to privacy.

It was in another part of the building which – I guess again, you know, we’ve always got those issues around, you know, we don’t want people blabbing about our business to all them other fellas, and I sort of felt that I could trust her that way (Woman, 53 years old).

I was a bit sceptic at the start because Aboriginal people being – like, everyone knows someone of someone. … Yeah, once I’d met ‘em spoke to ‘em, I was really, really at ease, yeah (Man, 32 years old).

Sometimes I felt like people knew if you were going to counselling, and when you’re down and out like that it’s big shame. Yeah, I don’t feel shamed now but when you’re down and you do feel that, at that time. At first I thought it would be that way, but she just greeted me (Woman, 43 years old).

The fear of being identified by their families and community was strongly linked to a feeling of shame. Health service staff identified shame as being a common concern for service users, and the AHWs’ and social worker’s excerpts below illustrate the significance that feelings of shame and the need for privacy have for service users.

It’s probably shameful for any Murri person to sit there and access a service, so the fact that they’re located just around the corner, it’s isolated and there’s a few different staff members that sit there, so they don’t always know that it’s about mental health issues (AHW: Focus group).

And it’s trust too because the families are very big and they don’t want everyone to know that they’ve been here to see you (Social Worker: Interview).

In the extracts above the importance of privacy is clearly highlighted, but once the service had been accessed a lot of fears were allayed. The availability of a range of Aboriginal and/or Torres Strait Islander health care providers at the Centre of Excellence has contributed to improvements in access for Aboriginal and Torres Strait Islander people to comprehensive primary health care. This is consistent with research that has demonstrated that a shared cultural identity enhances attendance at appointments [7, 10]. Given that shame is such a pervasive issue related to mental health [4, 9], one of the significant achievements of the Centre of Excellence was to sufficiently engage clients to access the service and experience a culturally safe environment. The three themes of responsiveness to community needs, trusted relationships and shared cultural identity, represent key features of the development and delivery of health care for social and emotional well-being within a framework of cultural safety.

## Conclusion

The experiences of health service users and staff of the addition of a psychologist and social worker to the primary health care service were overwhelmingly positive and access was significantly improved. Responsiveness to the needs of the Aboriginal and Torres Strait Islander community in the design, development and delivery of health care, trusted relationships and a shared cultural understanding were main features that contributed to the success of the expanded primary health care service. Improving social and emotional well-being is crucial to addressing the health inequalities experienced by Aboriginal and Torres Strait people. This study demonstrates the benefits of integrating culturally safe mental health services into primary health care.
